# Knowledge towards cervical cancer prevention and screening practices among women who attended reproductive and child health clinic at Magu district hospital, Lake Zone Tanzania: a cross-sectional study

**DOI:** 10.1186/s12885-018-4490-7

**Published:** 2018-05-16

**Authors:** Mabula M. Mabelele, John Materu, Faraja D. Ng’ida, Michael J. Mahande

**Affiliations:** 1 Department of Epidemiology and Biostatistics, Institute of Public Health, Kilimanjaro Christian Medical University College, Moshi, Tanzania; 2 Department of Community Health, Institute of Public Health, Kilimanjaro Christian Medical University College, Moshi, Tanzania

**Keywords:** Knowledge, Cervical cancer, Screening, Tanzania

## Abstract

**Background:**

Cervical cancer is a global leading cause of morbidity and mortality, attributable to the death of approximately 266,000 women every year. Majority (87%) of cervical cancer deaths occur in developing countries including Tanzania. Though knowledge of cervical cancer is an important determinant of women’s participation in prevention and screening for cervical cancer, little is known about this topic in Tanzania. This study aimed to determine the knowledge of cervical cancer prevention services and screening practices among women who attended Reproductive Child Health clinic at a district hospital in Lake Zone, Tanzania. This information is important to help designing appropriate interventions and scaling up cervical cancer control programs, hence accelerate the achievement towards Sustainable Development Goals.

**Methods:**

A cross-sectional study was conducted from March to June 2017, involving 307 women attending reproductive and child health clinic at Magu district hospital. A questionnaire adopted from the validated Cervical Cancer Awareness Measure was used to collect data from the study participants. Data was analysed using SPSS version 20. Descriptive statistics were summarized using frequencies and percentages for categorical variables while mean and standard deviation was used for continuous variables. Multivariable logistic regressions model was used to estimate Adjusted Odds ratio with 95% CI for factors associated with knowledge.

**Results:**

Knowledge of cervical cancer was low, where 82.7% of the women scored less than 50%. Majority (82.4%) were aware about cervical cancer. Secondary education or higher (OR = 7.77, 95% CI: 1.70-35.48) and “knowing someone who has ever had cervical cancer” (OR = 2.19, 95% CI: 1.16-4.13) were significantly associated with higher knowledge. Only 14.3% of participants practiced cervical cancer screening.

**Conclusions:**

Majority of women lack comprehensive knowledge of cervical cancer and only few utilize screening services. Strategies for awareness creation about cervical cancer may help to improve knowledge and utilization of cancer screening practices.

## Background

Cervical Cancer is a public health problem and a leading cause of mortality and morbidity among women [1, 2]. In 2012 there was an estimated 528,000 new cases and 266,000 deaths attributable to cervical cancer [2]. Majority (85%) of cervical cancer occurred in developing countries particularly in Sub-Saharan Africa (SSA). Tanzania being within the high risk region has an age standardized risk of developing cervical cancer of 54.4 per 100,000 person years compared with 5.5 and 4.4 per 100,000 person years for Australia – New Zealand and Western Asia respectively [2]. The high rates of cervical cancer can mainly be attributed to high prevalence of HPV infection and limited screening services [3]. Mortality due to cervical cancer varies from 27.6 per 100,000 in East Africa to less than 2 per 100,000 person years in Western Asia, Australia-New Zealand and Europe [2]. Apart from morbidity and mortality, cervical cancer and its treatment has significant social and economic consequences to cancer patients, families and countries at large as it leads to poor quality of life, increased treatment expenses and decreases productivity [4–8].

To prevent and control cancer, several initiatives have been implemented by the world health organization (WHO), government health ministries and non- governmental organizations. In Tanzania, the Ministry of Health Community Development, Gender, Elderly and Children (MoHCDGEC) is working to achieve goals of its Action and Strategic plan to control and prevent Cancer for 2016 - 2020. These goals include; 50% increase in proportion of patients detected with early stage cancer, to achieve 80% coverage of HPV vaccine among schoolgirls aged 9 – 13 years, 20% reduction in overall mortality from cancer, 60% of cancer patients accessing palliative care [9]. The National cervical cancer screening programme in Tanzania employs Visual Inspection with Acetic acid (VIA) test. This service is available free of charge at government owned district, regional and referral hospitals making up a total of 443 centres throughout the country. From the year 2012 – 2017 only 13% of the targeted women utilized cervical cancer screening services [].

To complement the government efforts, Medical Women Association of Tanzania (MEWATA) has been training health care workers, conducting mass sensitization and screening campaigns as well as treatment by Cryotherapy and Loop Electrosurgical Excision Procedure (LEEP) in many parts of Tanzania [10].

Despite the efforts to mitigate cervical cancer problem; the burden is still high and there are still a lot of challenges in cervical cancer control. These challenges are partly due to the economic constraints, competing priorities with other public health problems such as malaria, TB, HIV and lack of information and awareness [11–13].

Knowledge and awareness of cervical cancer is an important determinant of participation in cervical cancer prevention and control [14]. Several studies have been done to determine the knowledge of cervical cancer and screening practices among women but most studies have been conducted in urban setting making it difficult to generalize the findings to the general population. There is scant information about the knowledge of cervical cancer and screening practices of women in Tanzania. This information is important if cervical cancer control programs are to be successful.

This study aimed to determine cervical cancer knowledge and screening practices among women who attended reproductive and child health clinic (RCH) at Magu district hospital. The study provides information that could help in tailoring appropriate interventions and policy. It will also help to identify areas that need to be addressed by education programmes as well as establish a baseline that could be used to evaluate the effectiveness of future interventions.

## Methods

### Study design and setting

This was a health facility based cross-sectional study conducted at the reproductive and child health clinic (RCH) of the Magu district hospital from March to June 2017. Magu is district in Mwanza region just south of the great Lake Victoria. The district has a population of 299,759 which is served by one district hospital, division hospital, and seven dispensaries [15]. The district has a HIV prevalence of 4.7% and fertility rate of 4.7 both of which could be risk factors for cervical cancer [16, 17].

### Study population and sample size

This study involved all women of reproductive age (15 – 49 years) who attended RCH at Magu district hospital during the study period. Women who were critically ill and in need of immediate care and those who didn’t consent to participate were excluded from the study. A final sample size was 307 women which was calculated based on a previous study by Kileo and colleagues [18].

### Data collection methods and tools

Data was collected using a questionnaire adopted, with modifications, from the Cervical cancer awareness measure (Cervical- CAM) by UCL health behaviour research centre [19] and some of the items were developed from previous studies [20, 21]. Using the Swahili version of the questionnaire, we interviewed participants face to face and recorded their responses for open ended and close ended questions. To reduce bias interviewers were trained and emphasized to follow a standardized protocol. The questionnaire consisted of a set of questions including socio – demographic characteristics of the study participants, their awareness of cervical cancer, awareness of cervical cancer risk factors, symptoms, preventive measures, treatment options and screening practices. Both open ended and close ended questions were used to assess knowledge. Open ended questions required the participants to mention their response, while close ended questions required them to recognise the correct response from a list of alternatives. Open ended questions were presented before close ended questions. Each questionnaire was checked for completeness and scored for aspects of awareness of cervical cancer risk factors, symptoms, preventive measures and treatment options. The score was combined to generate a knowledge score for each participant.

### Statistical analysis

Data was checked for completeness, coded, cleaned and analysed using Statistical Package for Social Sciences (SPSS) version 20 (SPSS Inc. Chicago). Data from open ended questions was coded according to theme and quantitative analyses applied Descriptive statistics were summarized using frequencies and percentages for categorical variables while mean and standard deviation was used for continuous variables. Multivariable logistic regressions model was used to estimate Adjusted Odds ratio with 95% CI for factors associated with knowledge. Variables which showed significant association by chi square were included in the regression model and adjusted for each other to give adjusted odds ratio.

## Results

### Characteristics of study participants

Table 1 show the social demographic characteristics of the 307 participants who were recruited for this study. Their mean age was 27.04 (SD = 6.58) years. Majority, 195 (63.5%) had primary education, and were married 239 (77.9%). The mean parity was 2.8 (SD = 1.99) births. More than half of them 175 (57.0%) of the participants were peasants while only 8 (2.6%) had formal employment. Majority 234 (76.2%) of the participants were of the Sukuma tribe.

**Table 1:** Characteristics of study participants (*N* = 307)

Variables	Number	Percent
Age (years)^a^		
15 - 24	125	40.7
25 - 34	136	44.3
35 - 49	46	15.0
Education level		
Informal	42	13.7
Primary education	195	63.5
secondary or higher	70	22.8
Occupation		
Formal employment	8	2.6
Not employed (peasant, vendor etc.)	299	97.4
Area of residence		
Rural	126	41.0
Urban	181	59.0
Tribe		
Sukuma	234	76.2
Others	73	23.8
Marital status		
Single/divorced/widowed	63	20.5
Married/cohabiting	244	79.5
Type of marriage		
Monogamous	218	71.0
Polygamous	21	6.8
Missing^b^	68	22.1
Parity		
0 to 1	104	33.9
2 to 4	150	48.9
5 and above	53	17.2
Health insurance coverage		
Yes	41	13.4
No	266	86.6

^a^Mean age 27.04 (SD = 6.58) years

^b^68 participants appear as missing as they were not married

### Knowledge on cervical cancer

Majority 255 (83.1%) of the participants reported to have ever heard of cervical cancer disease. Of these 81 (31.8%) reported to have ever known someone who suffered from cervical cancer.

**Fig. 1:**
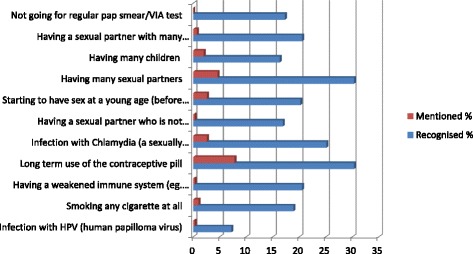
Proportions of participant who were able to “mention” or “recognize” cervical cancer risk factors

The proportions of participants who were able to mention or recognise various risk factors are shown in Fig. 1. Majority 253 (82.4%) of the participants were unable to mention any cervical cancer risk factor but 169 (55.0%) were able to recognise at least one from a list of eleven target risk factors. “Long term use of contraceptive pills” was the most frequently 24 (7.8%) mentioned risk factor while “infection with HPV”, “Having a sexual partner who is not circumcised” and “having weakened immunity” were least frequently mentioned (each by 0.3%, *n* = 1).

**Fig. 2:**
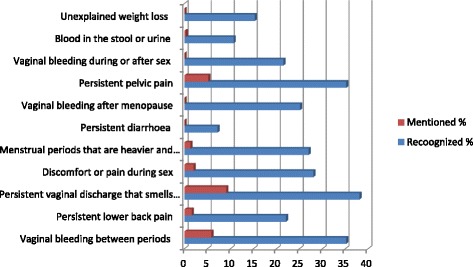
Proportions of participant who were able to “mention” or “recognize” cervical cancer symptoms

The proportions of participants who were able to mention or recognise various symptoms of cervical cancer are shown in Fig. 2. Majority 258 (84.0%) of participants were unable to mention any of the eleven target symptoms but when asked to recognize symptoms from a list, more than half 182 (59.3%) were able to recognize at least one target symptom. “Persistent vaginal discharge that smells unpleasant” was the most frequently recalled 28 (9.1%) and recognized 117 (38.1%) symptom. None of the participants mentioned; “Persistent diarrhoea”, “vaginal bleeding during or after sex” or “unexplained weight loss” as symptoms.

**Fig. 3:**
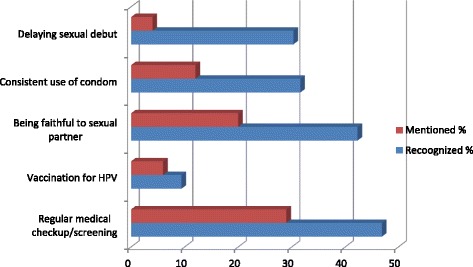
Proportions of participant who were able to “mention” or “recognize” cervical cancer preventive measures

The participants’ knowledge of cervical cancer preventive measures was as depicted by Fig. 3. Majority 258 (84%) of the participants were unable to mention any measure, but more than half 191 (62.2%) recognised at least one of the target preventive measures. “Regular medical check-up/screening” was the most 29 (9.4%) frequent response while “delaying sexual debut” was the least 6 (2.0%).

Majority 194 (63.2%) of the participants report to have ever heard of cervical cancer screening while 121 (39.4%) were aware of the existence of a national cervical cancer screening program in Tanzania. When asked about the recommended age to start screening in Tanzania; most 258 (84.0%) didn’t know, 34 (11.1%) said at the age of eighteen years, while only two (0.7%) stated “correctly” 30 years. Only 24 (7.8%) of the participants were aware of Human Papilloma Virus (HPV) vaccine and none of the age at which the vaccine is administered.

**Fig. 4:**
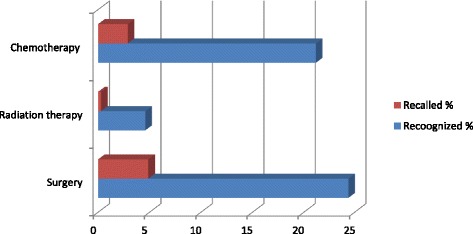
Proportions of participant who were able to “mention” or “recognize” cervical cancer treatment options

Knowledge on cervical cancer treatment options among participants was as illustrated in Fig. 4. Only 20 (6.5%) participants were able to mention at least one treatment option and merely one third 103 (33.6%) were able to recognize at least one treatment option from a list. Surgery was the most frequently recalled 15(4.9%) and recognized by 75 (24.4%) treatment. Only one participant (0.3%) mentioned radiation therapy as treatment option for cervical cancer.

Scores for cervical cancer awareness, awareness of risk factors, symptoms, prevention measures, cervical cancer screening, HPV vaccine and treatment options were combined to give a comprehensive knowledge score. Recognition scores were used for this purpose. The scores ranged from 0 to 83.3% with a median score of 16.67%. Majority 254 (82.7%) of the participants scored less than 50% and were considered to have inadequate knowledge on cervical cancer. Only 53 (17.3%) had adequate knowledge as they scored 50% or above.

### Factors associated with knowledge on cervical cancer

Association between study participants’ demographic characteristics and adequacy of their knowledge on cervical cancer was as shown in Table 2. Multi variable logistic regression was performed with variables that showed significant association by chi-square being included in the model. Participants’ education level especially secondary or higher (AOR = 7.77, 95% CI: 1.70 - 35.48) and ‘knowing somebody who has ever had cervical cancer’ (AOR = 2.19, 95% CI: 1.16 - 4.13) were independently, significantly associated with knowledge on cervical cancer. Formal employment, being married or cohabiting, monogamous type of marriage, higher parity (grand multiparty) and having health insurance coverage increased the likelihood of adequate cervical cancer knowledge but these were not statistically significant.

**Table 2:** Socio demographic factors associated with cervical cancer knowledge (*N* = 307)

	Knowledge		Crude	Adjusted
Variables	Adequate n(%)	Inadequate n(%)	OR (95% CI)	AOR^a^ (CI)
Age (years)^b^				
15 - 24	20(16.0)	105(84.0)	1.0	
25 - 34	24(17.6)	112(82.4)	0.78(0.33-1.87)	
35 - 49	9(19.6)	37(80.4)	0.88(0.33-2.06)	
Education level				
Informal	2(4.8)	40(95.2)	1.0	1.0
Primary education	29(14.9)	166(85.1)	3.49(0.80-15.23)	3.32(0.76-14.58
Secondary or higher	22(31.4)	48(68.6)	9.17(2.03-41.37)	7.77(1.70-35.48)
Occupation				
Formal employment	2(25.0)	6(75.0)	1.62(0.32-8.26)	
Not employed	51(17.1)	248(82.9)	1.0	
Area of residence				
Rural	19(15.1)	107(84.9)	1.0	
Urban	34(18.8)	147(81.2)	0.77(0.42-1.42)	
Tribe				
Sukuma	40(17.1%)	194(82.9)	1.0	
Other	13(17.8)	60(82.2)	1.05(0.53-2.09)	
Marital status				
Single/divorced/widowed	6(9.5)	57(90.5)	1.0	
Married/cohabiting	47(19.3)	197(80.7)	2.27(0.92-5.71)	
Type of marriage				
Monogamous	44(20.2)	174(79.8)	1.0	
Polygamous	2(9.5)	19(90.5)	0.42(0.93-1.86)	
Missing^c^	7(10.3)	61(89.7)	0.45(0.19-1.06)	
Parity				
0 to 1	18(17.3)	86(82.7)	1.0	
2 to 4	24(16.0)	126(83.0)	0.91(0.47-1.78)	
5 and above	11(20.8)	42(79.2)	1.25(0.54-2.89)	
Health insurance coverage				
No	45(16.9)	221(83.1)	1.0	
Yes	8(19.5)	33(80.5)	1.19(0.52-2.74)	
Know anyone who has ever had cervical cancer				
No	30(13.3)	196(86.7)	1.0	1.0
Yes	23(28.4)	58(71.6)	2.59(1.40-4.80)	2.19(1.156-4.13)

^a^Variables included in the multivariable logistic regression model adjusted for one another

^b^Mean age 27.04 (SD = 6.58) years

^c^68 appear as missing as theses participants were not married

### Cervical cancer screening practices

Based on self-reported screening practices of the participants, only 44 (14.3%) of women reported to have ever been screened for cervical cancer. Screening “rate” was higher (17.8%) among women age thirty and above, as compared to (12.5%) among their younger counterparts. Of the women who had ever screened, majority 28 (63.6%) rarely reported about screening (i.e. less than once in three years).

## Discussion

In this study, we found majority (83.1%) of women were aware of cervical cancer, this was comparable to 78.7% that was reported among Ethiopian women [20] but higher than 29% reported in Kenyan [22]. This difference is probably due to the in time lag between this study and the latter and possible educational interventions that may have occurred during that time lag. Though awareness alone is not enough, this level of awareness is a step in the right direction to improve upon.

We found only 17.6% of women were able to mention at least one cervical cancer risk factor which is the lowest compared with 31.0% among Ethiopian women [20] and 35% among British women [23]. This difference is probably due to higher education levels of participants and better cervical cancer awareness programs in the latter countries.

Interestingly, this study found “long term use of contraceptive pills” as the most frequently mentioned 24 (7.8%) and recognized 93 (30.3%) risk factor for cervical cancer. Our finding is in contrast with previous studies which reported sexual behaviour related factors as the most frequently identified risk factors [20, 23]. This could possibly be a result of genuine awareness of this particular risk factor or could be a result of misconception as women have been known to disproportionately associate birth control pills with lots of side effects such cancer and infertility [24]. However, this requires further studies to substantiate.

Although majority of women were aware of cervical cancer, only 53 (17.3%) women had adequate knowledge on cervical cancer. This was lower compared with the 31.0% which was previously reported among Ethiopian women [20]. The observed difference could be due to slight differences in the study tools, both studies showed low knowledge about cervical cancer. Low knowledge could be due to low coverage of cancer awareness initiatives in African countries. This calls for action to improve the knowledge on cervical cancer as it is a determinant of screening utilisation and an important component of cervical cancer prevention.

This study found education level and “knowing someone who has ever had cervical cancer” to be significant predictors of adequate cervical cancer knowledge, consistent with other studies [20, 23]. This consistency emphasizes the influence of formal education and close experience in understanding health related issues. This reflect that a multi-sectoral approach (especially education sector) would be more effective in prevention and control of diseases like cervical cancer.

We found a low self-reported screening practice, where only 14.3% of all participants reported to have ever screened for cervical cancer. Among women age 30 and above only 17.8% had ever been screened. Previous studies conducted in Tanzania and other parts of SSA have also reported low rates ranging from 6 to 21% [18, 21, 22, 25, 26]. This may reflect the low coverage and utilisation of screening services in Tanzania and other SSA countries. Since screening is an integral part of early cancer diagnosis and subsequently better prognosis, there is a need for efforts to improve coverage and utilisation of screening services. This can be achieved by identifying and addressing barriers to cervical cancer screening.

### Strengths and limitations of the study

This study provides important information but may be subject to limitations. This study was hospital-based, recruiting only women attending RCH clinic and may not be representative of the whole community. Also the study relied on self-reported screening practices which may be subject to reporting bias or self-desirability bias.

## Conclusion

Majority of women lack comprehensive knowledge of cervical cancer and only few utilize screening services. Strategies for awareness creation about cervical cancer may help to improve knowledge and utilization of cancer screening practices.

## Abbreviations

Cervical – CAM: Cervical Cancer Awareness Measure
GLOBOCAN: Global Burden of Cancer
HCW: Health Care Workers
HPV: Human Papilloma Virus
LEEP: Loop Electrosurgical Excision Procedure
MEWATA: Medical Women Association of Tanzania
MoHCDGEC: Ministry of Health Community Development, Gender, Elderly and Children
VIA: Visual Inspection with Acetic acid
